# High rates of breast conservation for large ductal and lobular invasive carcinomas combining multimodality strategies

**DOI:** 10.1038/sj.bjc.6604229

**Published:** 2008-02-05

**Authors:** M A Bollet, A Savignoni, J-Y Pierga, M Lae, V Fourchotte, Y M Kirova, R Dendale, F Campana, B Sigal-Zafrani, R Salmon, A Fourquet, A Vincent-Salomon

**Affiliations:** 1 Department of Radiation Oncology, Institut Curie, Paris cedex 05, Paris 75248, France; 2 Department of Biostatistics, Institut Curie, Paris cedex 05, Paris 75248, France; 3 Department of Medical Oncology, Institut Curie, Paris cedex 05, Paris 75248, France; 4 Department of Pathology, Institut Curie, Paris cedex 05, Paris 75248, France; 5 Department of Surgery, Institut Curie, Paris cedex 05, Paris 75248, France

**Keywords:** breast cancer, neoadjuvant, chemotherapy, radiotherapy, multimodality

## Abstract

The literature reports low rates of breast conservation after neoadjuvant chemotherapy for operable breast cancers not amenable to initial breast-conserving surgery. This study aims to compare the outcome of lobular *vs* ductal carcinomas after neoadjuvant chemotherapy. Between 1989 and 1999, 750 patients with clinical stage II/IIIA ductal (672) or lobular (78) invasive breast carcinomas were treated at the Institut Curie with primary anthracycline-based polychemotherapy followed by either breast conservation (surgery and/or radiotherapy) or mastectomy. Median follow-up was 10 years. Clinical response to primary chemotherapy was significantly worse for lobular than for ductal carcinomas (47 *vs* 60%; *P*=0.04), but only histological grade remained predictive in multivariate analysis. Breast conservation was high for both ductal and lobular carcinomas (65 and 54%; *P*=0.07), due, in part, to the use of radiotherapy, either exclusive or preoperative, for respectively 26 and 40% of patients. The lobular type had no adverse effect, neither on locoregional control nor on overall survival, even in the group of patients treated with breast conservation.

The standard treatment of operable breast adenocarcinomas too large to be amenable to breast-conserving surgery is mastectomy with axillary lymph node clearance. Several randomised studies have compared neoadjuvant with adjuvant chemotherapy. In these studies, the locoregional treatment could be either surgery or radiotherapy or a combination of both. There was no significant difference in terms of locoregional control and overall (OS) or disease-free survivals (Mauri *et al*, 2005). The principal clinical benefit of a neoadjuvant medical treatment was, in the case of good clinical response, avoiding mastectomy. However, data regarding the specific behaviour of invasive lobular carcinomas (ILC), a histological subtype of breast cancers representing 5–15% of all breast cancer cases, are still scarce in this therapeutic setting. Lobular carcinomas are characterised by a specific morphology with discohesive small cells usually associated with estradiol receptor (ER), progesterone receptor (PR) positivity and with a low proliferation rate (Sastre-Garau *et al*, 1996). These factors are now well-established predictive markers of poor response to neoadjuvant chemotherapy (Pierga *et al*, 2003). We, and others, have previously shown that ILC are often diagnosed as larger tumours than invasive ductal carcinomas (IDC) (Sastre-Garau *et al*, 1996), and for which a neoadjuvant treatment could be chosen to increase the rate of conservative surgery. This study aims to compare the outcome of lobular *vs* ductal carcinomas under neoadjuvant chemotherapy and to report their locoregional control.

## MATERIALS AND METHODS

Between January 1989 and December 1999, a total of 803 patients, with operable, clinical stage II/IIIA, either ductal or lobular invasive breast carcinoma, not amenable to initial breast-conserving surgery, were treated at the Institut Curie with primary chemotherapy. Out of them, 750 had an initial core-needle biopsy before the start of treatment and will be studied here – 78 patients with lobular invasive carcinoma and 672 with ductal invasive carcinomas. The other 53 had initial fine-needle aspiration and underwent biopsy after the start of the chemotherapy. Median follow-up was 117 months (10–188). Histological classification was made according to the WHO criteria. Histological grading was performed according to the Scarff Bloom and Richardson method (Bloom and Richardson, 1957). Positivity to ER and PR was determined by biochemistry. Hormonal receptors (HR) were considered positive when either ER or PR was positive. All slides of lobular carcinomas were retrospectively reviewed. The E-cadherin expression was assessed by immunohistochemistry.

Treatment decisions were not adapted to the histological type. The following information regarding the treatment details apply both to lobular and ductal carcinomas. No patient received neoadjuvant hormonal therapy. All patients received a median of four (range: 1–6) cycles of neoadjuvant chemotherapy. Chemotherapy between 1989 and 1995, consisted of FAC with 5-Fluorouracil (F) on days 1, 3, 5 and 8, adriamycin (A) on days 1 and 8, and cyclophosphamide (C) on days 1 and 8 (Pierga *et al*, 2003). It was subsequently simplified according to a more classical regimen with adriamycin or epirubicin (E), cyclophosphamide and/or fluorouracil every 21 days for six cycles (FAC, FEC, AC or EC) (French Adjuvant Study Group, 2001).

Response to primary chemotherapy was assessed clinically as reported by Pierga *et al* (2003), according to the International Union against Cancer Criteria. Complete response was defined as the total resolution of the breast mass and regional lymph adenopathy as determined by physical examination; partial response was defined as 50% or greater reduction in the product of the two largest perpendicular dimensions of the breast mass and regional adenopathy; minor response was defined as less than a 50% reduction in the product of the two largest perpendicular dimensions. Stable disease was defined as no measurable change in the product of the two largest perpendicular dimensions, and progressive disease was defined as an increase of at least 25% in the product of the two largest perpendicular dimensions. Clinical response was dichotomised in two groups: <50 and ⩾50%.

Local treatments consisted in breast surgery and/or radiotherapy. Whenever feasible, it consisted in tumorectomy followed by radiotherapy. When the tumour did not become amenable to conservative surgery, the decision was usually made to perform a mastectomy, followed in most cases by radiotherapy. However, some patients, because of a desire to conserve their breasts, received radiotherapy as first (followed by either tumorectomy or mastectomy) or exclusive local treatment. In other instances, this sequence of preoperative or exclusive radiotherapy was chosen for tumours with very good response to chemotherapy. Radiotherapy delivered a mean dose of 51.4 Gy (s.d. 2.5 Gy) in 2 Gy fractions to the breast, using either standard or lateral decubitus techniques (Campana *et al*, 2005), or to the chest wall. A mean dose of 45 Gy (s.d., 2 Gy) in 23–25 fractions was usually delivered to the internal mammary chain and the supraclavicular area with the addition of an axillary irradiation when there was an important axillary involvement or in the absence of an axillary lymph node dissection. In the case of breast–conserving treatments, a boost was delivered to the tumour or to the tumorectomy bed by either external beam radiotherapy or brachytherapy. The mean total dose was 64 Gy (s.d., 7.6 Gy) to the tumorectomy bed for postoperative radiotherapy and 73 Gy (s.d., 7.6 Gy) to the tumour in the case of exclusive radiotherapy. Patients were followed up clinically every 6 months for the first 5 years and annually thereafter. Mammograms and/or ultrasound scans were performed annually.

### Statistics

All studied factors are reported in the relevant Tables.

Differences between groups were analysed by χ^2^ tests for categorical variables and Student's *t*-test for continuous variables.

Univariate logistic regressions were performed to identify predictive factors of clinical response (⩾50%) and to estimate crude odds ratios (OR). Multivariate logistic regression analyses permitted to estimate the OR of variables independently associated with clinical response.

Survival data were defined as the time from diagnosis of breast cancer until the occurrence of event, that is, locoregional recurrence (LRR) defined as any recurrence occurring in the treated breast or in the ipsilateral chest wall or regional lymph node-bearing areas (internal mammary chain, axilla and supraclavicular area) and death (OS). Recurrence-free and alive patients were censored at the date of their last known contact. The OS and LRR rates were calculated by the Kaplan–Meier method, and groups were compared using a log-rank test. Multivariate analysis was carried out to assess the relative influence of prognostic factors on locoregional control, using the Cox stepwise procedure after stratification on the local treatment (exclusive/preoperative radiotherapy *vs* postoperative radiotherapy) (Cox and Oakes, 1984). Missing values (tumour grade, receptor levels and mitotic index) were coded as separate categories of the variable and retained in the model.

Significance level was 0.05. Analyses were performed using Splus 2000 software (Mathsoft Inc., Seattle, WA, USA).

## RESULTS

### Patients' and tumours' characteristics according to the histological type of invasive carcinoma

The comparison of the 78 patients with lobular invasive carcinoma with the 672 with ductal invasive carcinomas is summarised in Table 1.

Patients diagnosed with lobular invasive carcinomas were significantly older than patients with ductal carcinomas. Lobular cancers were significantly larger (median size of 50 *vs* 40 mm; *P*<10^−3^) with more HRs positive (HR+; 87 *vs* 73%; *P*=0.02) and lower histological grades than ductal carcinomas (grade 1; 41 *vs* 12%; *P*<10^−3^).

### Response to primary chemotherapy and local treatment according to histological type

Clinical response to primary chemotherapy was significantly better for ductal invasive carcinomas than for lobular (⩾50% of clinical response in 60% for ductal *vs* 47% for lobular; *P*=0.04; Table 2). The rates of breast-conserving treatments for lobular carcinomas was lower than that for ductal carcinomas, but the difference was not statistically significant (54% (42 out of 78) *vs* 65% (434 out of 672); *P*=0.07) and more patients with lobular carcinomas received local treatment with either exclusive radiotherapy or preoperative radiotherapy compared with ductal carcinomas (40 (31 out of 78) *vs* 26% (178 out of 672); *P*=0.02). If we look at patients who had surgery, excluding patients with radiotherapy alone as local treatment, there were significantly fewer breast conservations for lobular than for ductal invasive carcinomas: 24 out of 78 (31%) *vs* 333 out of 672 (50%), *P*=0.001. Rates of pathological complete response (for all patients who had undergone surgery) were respectively 9% (40 out of 457, 118 unknown) for ductal and 8% (4 out of 50, 10 unknown) for lobular (*P*=0.94).

Other factors of good clinical response found in univariate analysis (Table 3) were clinical T2 *vs* T3, HR+ *vs* HR− and high histological grade *vs* low and intermediate. In multivariate analysis, only high grade was significantly associated with a higher rate of clinical response.

### Comparison of patients who underwent breast conservation with either preoperative/exclusive radiotherapy or postoperative radiotherapy

Among patients who received breast-conserving treatments, those with preoperative/exclusive radiotherapy had tumours that were significantly more often clinically T3 (23 *vs* 15%; *P*=0.03), lobular (15 *vs* 5%; *P*<10^−3^) and responded clinically less to primary chemotherapy (clinical response ⩾50% respectively 63 *vs* 78%; *P*<10^−3^) than those with postoperative radiotherapy (Table 4).

### Survivals

Overall survival rates at, respectively, 5 and 10 years were 81 (95% CI, 78–84%) and 66% (95% CI, 62–70%).

Locoregional control rates at, respectively, 5 and 10 years were 83 (95% CI, 81–86%) and 75% (95% CI, 71–78%) but, when considering only patients treated with breast conservation, 81 (95% CI, 77–85%) and 71% (95% CI, 67–76%).

### Prognostic factors

#### For locoregional control

In univariate analysis, factors that were found to be associated with a higher locoregional recurrence rate were young age, cN1, HR− and having undergone a breast-conserving treatment (Table 5). The histological subtype classification (lobular *vs* ductal) was not found to be prognostic for locoregional control, with values at 10 years being 73 and 75%, respectively (RR=1 (0.6–1.7); *P*=0.9).

On forward stepwise multivariate analysis (Cox model) stratified on local treatment, only the young age (<40 years) remained statistically significant.

When considering only patients treated with breast conservation (Table 6), the only factors found in univariate analysis to be associated with a higher locoregional recurrence rate were young age and treatment with preoperative or exclusive radiotherapy. The histological subtype classification (lobular *vs* ductal) was not found to be prognostic for locoregional control with, respectively, at 10 years, a locoregional control of 62 and 72% (RR=1.5 (0.9–2.6); *P*=0.12).

On multivariate analysis (Cox model) stratified on local treatment, only young age (<40 years) remained statistically associated with worse locoregional control.

#### For overall survival

In univariate analysis, factors that were found to be associated with a lower overall survival rate were cT3, cN1, HR−, histological grade 2 and 3 and mitotic index grade 2 and 3 (Table 7). The histological subtype classification (lobular *vs* ductal) was not found to be prognostic for overall survival with, respectively, at 10 years, a rate of 72% (95% CI, 62–84%) and 65% (95% CI, 61–69); RR=0.84 (0.55–1.30); *P*=0.44.

On forward stepwise multivariate analysis (Cox model) stratified on local treatment, cT3, cN1, HR−, histological grade 2/3 and absence of clinical response were statistically significant.

## DISCUSSION

This retrospective study spans a period of 10 years with patients treated between 1989 and 1999. The follow-up is long with a median at 10 years and makes the study of locoregional survival relevant. The tumour characteristics of lobular carcinomas were those already found by Sastre and co-workers and others (Sastre-Garau *et al*, 1996; Cristofanilli *et al*, 2005). Patients diagnosed with lobular invasive carcinomas were significantly older (Sastre-Garau *et al*, 1996) than patients with ductal tumours. Lobular cancers were significantly larger (Sastre-Garau *et al*, 1996; Cristofanilli *et al*, 2005; Tubiana-Hulin *et al*, 2006) with more HRs (Sastre-Garau *et al*, 1996; Mathieu *et al*, 2004; Cristofanilli *et al*, 2005; Tubiana-Hulin *et al*, 2006) and lower histological (Sastre-Garau *et al*, 1996; Tubiana-Hulin *et al*, 2006) or nuclear (Cristofanilli *et al*, 2005; Tubiana-Hulin *et al*, 2006) grade than ductal carcinomas.

Because of the frequent use of either exclusive or preoperative radiotherapy, the study of pathological response in our series was not relevant. Bearing in mind the mandatory caution in interpreting clinical response (Bollet *et al*, 2007), we found that the response to primary chemotherapy was significantly better for ductal invasive carcinomas than for lobular (⩾50% of clinical response in 60% for ductal *vs* 47% for lobular; *P*=0.04), confirming the notion developed by others that lobular carcinomas responded less than ductal carcinomas to neoadjuvant chemotherapy (Mathieu *et al*, 2004; Cristofanilli *et al*, 2005; Tubiana-Hulin *et al*, 2006; Wenzel *et al*, 2006; Katz *et al*, 2007). However, in the present study, in multivariate analysis, only high grade was significantly associated with a higher rate of clinical response. This result is therefore in agreement with the Nomograms's design proposed online, which does not take into account the histological type of the tumour when deciding on the therapeutic option (Rouzier *et al*, 2005b). There is room to better define biological predictive factors of response to neoadjuvant chemotherapy to screen patients who would benefit most from this therapeutic option (Sotiriou *et al*, 2002; Chang *et al*, 2005; Gianni *et al*, 2005; Rouzier *et al*, 2005a; Pierga *et al*, 2007). Lobular carcinomas had reduced clinical responses to neoadjuvant chemotherapy, while responses to breast-conserving treatments were fewer, although not significantly (54% (42 out of 78) *vs* 65% (434 out of 672); *P*=0.07). The lower rate of breast conservation in lobular *vs* ductal invasive carcinoma could also be due to the larger size of ILC. The most striking data are the high rate of breast conservation for both ductal and even more so for lobular carcinomas when compared to the rates in the literature that range, respectively, between 30–48 and 16–31% (Mathieu *et al*, 2004; Cristofanilli *et al*, 2005; Tubiana-Hulin *et al*, 2006). This is explained in our series by the use of either exclusive or preoperative radiotherapy, particularly for large tumours with a poor response to neoadjuvant chemotherapy and, therefore, more frequently for lobular (40%) than for ductal (26%) carcinomas. Because of the retrospective nature of this study, we could not report the reason for the choice of performing preoperative or exclusive radiotherapy. However, as one can see from Table 2, the proportion of patients who received radiotherapy immediately after primary chemotherapy and who actually went on to have successful breast-conserving treatments was 80 and 84%, respectively, for IDC and ILC.

Despite being associated with large tumour size and less clinical response to primary chemotherapy, lobular subtype was not found associated with either worse locoregional control, even in the subgroup of only patients who had been treated with breast conservation, or worse overall survival. It should, however, be noted that in this series, patients treated with exclusive or preoperative radiotherapy fared worse in terms of locoregional control than others, perhaps also because of a selection of patients with poor prognosis. This opens the debate as to whether radiotherapy could not play a role in increasing the rate of breast-conserving treatments for some invasive carcinomas, as it is an important part of the treatment of large breast cancers (Whelan *et al*, 2000; Clarke *et al*, 2005) and has been shown to achieve complete clinical response (6–41%) with doses compatible with planned secondary surgeries (Calitchi *et al*, 1991; Scholl *et al*, 1994; Broet *et al*, 1999; Bollet *et al*, 2006). At the Institut Curie, the S6 trial randomised premenopausal women between chemotherapy and radiotherapy as an initial treatment for large breast cancers (Scholl *et al*, 1994). There was no difference in the 10-year OS (Broet *et al*, 1999). Out of 69 tumours still palpable after four cycles of FAC (5-fluorouracil, adriamycine and cyclophosphamide), 42 (61%) achieved complete clinical response after 54 Gy. In contrast with chemotherapy (Vincent-Salomon *et al*, 2004; Andre *et al*, 2005), which is more effective in proliferative tumours, the effect of radiotherapy does not seem to depend on the degree of proliferation (or on the histological grade) (Remvikos *et al*, 1993; Rozan *et al*, 1998; Clarke *et al*, 2006). Radiotherapy could thus theoretically play a role in increasing the rate of breast conservation for tumours that fail to sufficiently shrink with primary chemotherapy because of low proliferation, as in the case of lobular carcinomas. Another study performed at the Institut Curie showed a similar rate of pathological complete response with preoperative concurrent chemoradiotherapy for lobular and for ductal invasive carcinomas (31 *vs* 22%; *P*=0.5; Bollet *et al*, 2006). Ring *et al* (2003) found, in a retrospective study comparing surgery (either breast-conserving or mastectomy) and exclusive radiotherapy for women who achieved clinical complete responses after primary chemotherapy, a nonsignificant trend toward increased locoregional-only recurrence for the no surgery group. However, they also found that patients achieving both clinical and ultrasound complete responses, and not undergoing surgery, had a low (8%) rate of locoregional recurrence at 5 years. Additionally, MRI has also proven to be useful in the assessment of the tumour response to chemotherapy (Thibault *et al*, 2004; Warren *et al*, 2004; Partridge *et al*, 2005; Segara *et al*, 2007). This good value of MRI in the evaluation of tumour response should also apply to ILC as, even though ILC is still a challenge for MRI (Kinkel and Hylton, 2001), the addition of MRI to mammography still increases the sensitivity in the detection of ILC (Gilles *et al*, 1994; Sittek *et al*, 1998) and, most importantly in the case of the response evaluation, the volumetric assessment is improved (Boetes *et al*, 2004; Fabre Demard *et al*, 2005; Caramella *et al*, 2007; Mann *et al*, 2008). This raises the question of whether patients who achieve complete clinical and imaging response could in some cases entirely avoid surgery. This issue, needless to say, would need to be properly assessed in a prospective randomised trial. Additionally, to really benefit from higher rates of breast conservation, one needs to closely evaluate the cosmetic effects of this therapeutic approach but which could not be assessed in our study. One particular concern is indeed that it is associated with worse cosmetic results, because they deteriorate with the radiotherapy total dose (whole breast+boost; Vrieling *et al*, 1999), with chemotherapy (Vass and Bairati, 2005), and when radiotherapy is given pre- rather than post-operatively, both in the case of standard (Durand *et al*, 1987) or oncoplastic (Clough *et al*, 2003) surgeries.

In conclusion, our series confirmed that lobular carcinomas demonstrate a lower response rate to neoadjuvant chemotherapy than ductal carcinomas, but that in multivariate analysis only low histological grades remained predictive of poor response to chemotherapy. The breast conservation in our series was higher than the one expected from the literature for both histological types. The most obvious explanation was the frequent use of either preoperative or exclusive radiotherapy after chemotherapy, especially for tumours with a poor response to chemotherapy and therefore for lobular carcinomas. The lobular type had no adverse effect on locoregional control even in the group of patients treated with breast conservation. Further studies are still needed to evaluate the exact role of preoperative/exclusive radiotherapy after neoadjuvant chemotherapy, not only in terms of efficacy but also of tolerance and long-term cosmetic effects.

## Figures and Tables

**Table 1:** Tumour and treatment characteristics according to the histological type of the invasive breast carcinoma

	**Ductal (*N*=672)**		**Lobular (*N*=78)**		
**Total**	***N***	**%**	***N***	**%**	***P***
*Age (DM*=*0*)					<10^−2^
Median (min–max) in years	46 (24–70)		49 (39–68)		
					
*Menopause (DM*=*0*)					0.88
Yes	167	25	20	26	
No	505	75	58	74	
					
*Clinical T stage (DM*=*0*)					0.03
T2	506	75	50	64	
T3	166	25	28	36	
					
*Clinical N stage (DM*=*0*)					0.16
N0	411	61	54	69	
N1	261	39	24	31	
					
*Tumour clinical maximal diameter (DM*=*0*)					<10^−3^
Median (min–max) in mm	40 (20–70)		50 (25–70)		
					
*Histological grade (DM*=*4*)^a^					<10^−3^
1	82	12	32	41	
2	332	50	30	38	
3	210	31	7	9	
					
*Mitoses per 10 HPF (DM*=*72*)					<10^−3^
(0–10)	129	21	53	80	
(11–21)	182	30	7	11	
⩾22	291	48	6	9	
					
*Hormonal receptors (DM*=*118*)					0.02
ER+ and/or PR+	412	73	54	87	
ER−/PR−	158	28	8	13	

HPF=high-power field; DM=data missing.

Menopause defined, for women with intact uterus, as 2 years of amenorrhea and for the others, biologically.

Percentages were rounded, so, when added, the sum may not be exactly 100.

a Fifty-three tumours (44 ductal and 9 lobular) could not be evaluated.

**Table 2:** Clinical responses to neoadjuvant chemotherapy

	**Ductal (*N*=672)**		**Lobular (*N*=78)**		
**Total**	***N***	**%**	***N***	**%**	***P***
*Clinical response to primary chemotherapy (DM*=*7*)					0.04
⩾50%	397	60	37	47	
<50%	268	40	41	53	
					
*Local treatment (DM*=*0*)					0.01
Tumorectomy followed by RT	292	43	16	21	
Mastectomy followed by RT	160	24	25	32	
Mastectomy alone	42	6	6	8	
RT followed by tumorectomy	45	7	8	10	
RT followed by mastectomy	36	5	5	6	
RT alone	97	14	18	23	

DM=data missing; RT, radiorherapy.

Local treatments.

Percentages were rounded, so, when added, the sum may not be exactly 100.

**Table 3:** Predictive factors of clinical response (>50%) according to univariate and multivariate analyses

			**Univariate analysis**		**Multivariate analysis**	
	***N***	**CR (%)**	***P***	**RR (IC 95%)**	***P***	**RR (IC 95%)**
*Age*						
>40	548	58		1		
⩽40	138	55	0.6	0.9 (0.6–1.3)		
						
*Menopause*						
No	512	56				
Yes	174	61	0.3	1.2 (0.8–1.7)		
						
*Clinical tumour stage*						
cT2	515	60				
cT3	171	51	<0.05^a^	0.7 (0.5–0.99)	0.12	0.7 (0.5–1.1)
						
*Clinical nodal status*						
cN0	425	56				
cN1	261	60	0.3	1.2 (0.9–1.6)		
						
*Histological type*						
Ductal	617	59				
Lobular	69	43	0.02	0.5 (0.3–0.9)	0.23	0.7 (0.4–1.3)
						
*Hormonal receptors* b						
HR+	433	55				
HR−	144	65	0.03	1.5 (1.0–2.3)	0.09	1.4 (0.9–2.1)
						
*Histological grade*						
Grade 1	113	42		1		1
Grade 2	360	59	0.003	1.9 (1.3–3.0)	0.02	1.8 (1.1–3.0)
Grade 3	213	63	<10^−3^	2.3 (1.4–3.7)	0.02	2.0 (1.1–3.4)

ER=estradiol receptor; HR=hormonal receptor; PR=progesterone receptor.

a Factors entered in the multivariate analysis.

b HR+ if ER+ and/or PR+.

**Table 4:** Comparison of patients treated with breast conserving treatment with either exclusive/preoperative radiotherapy or postoperative radiotherapy

	**Exclusive or preoperative radiotherapy (*N*=168)^a^**		**Postoperative radiotherapy (*N*=308)**		
**Total**	***N***	**%**	***N***	**%**	***P***
*Menopause (DM*=*0*)					0.44
No	121	72	232	75	
Yes	47	28	76	25	
					
*Clinical T stage (DM*=*0*)					0.03
T2	129	77	262	85	
T3	39	23	46	15	
					
*Clinical N stage (DM*=*0*)					0.21
N0	105	63	210	68	
N1	63	37	98	32	
					
*Histological type (DM*=*0*)					<10^−3^
Ductal	142	85	292	95	
Lobular	26	15	16	5	
					
*Clinical response to primary chemotherapy*					<10^−3^
⩾50%	104	63	239	78	
<50%	60	37	67	22	
					
*Hormonal receptors (DM*=*71*)					0.24
ER+ and/or PR+	99	68	192	74	
ER−/PR−	46	32	68	26	

DM=data missing; ER=estradiol receptor; HR=hormonal receptor; PR=progesterone receptor.

a 53 preoperative radiotherapy and 115 exclusive radiotherapy.

**Table 5:** Univariate and multivariate analyses of prognostic factors for locoregional recurrence-free survival

			**Univariate analysis**		**Multivariate analysis**	
	***N***	**10-year LRRFS (%) (IC 95%)**	***P* a**	**RR (IC 95%)**	***P* b**	**RR (IC 95%)**
Age (continuous variable)			0.015	0.97 (0.96–1.00)		
						
*Age (dummy variable)* c			<10^−6^		<10^−3^	
>40	597	79 (75–82)		1		1
⩽40	153	60 (51–70)		2.2 (1.6–3.1)		2.3 (1.6–3.2)
*Menopause*			0.5			
No	563	73 (69–78)		1		
Yes	187	79 (73–86)		0.9 (0.6–1.3)		
						
*Clinical tumour stage* c			0.35			
cT2	556	74 (69–78)		1		
cT3	194	78 (72–86)		0.8 (0.6–1.2)		
						
*Clinical nodal status* c			0.33			
cN0	465	76 (71–80)		1		
cN1	285	74 (68–80)		1.2 (0.8–1.6)		
						
*Histological type*			0.9			
Ductal	672	75 (71–79)		1		
Lobular	78	73 (62–86)		1 (0.6–1.7)		
						
*Hormonal receptors* c			0.05		0.02	
HR+	466	75 (71–80)		1		1
HR−	166	69 (62–78)		1.4 (1.0–2.0)		1.7 (1.2–2.5)
						
*Histological grade*			0.34			
Grade 1	114	82 (75–90)		1		
Grade 2	362	73 (68–79)		1.3 (0.8–2.0)		
Grade 3	217	73 (67–81)		1.4 (0.9–2.4)		
						
*Mitotic index*			0.48			
Grade 1	182	79 (72–87)		1		
Grade 2	189	77 (70–84)		1.2 (0.7–1.9)		
Grade 3	297	75 (69–81)		1.3 (0.8–2.0)		
						
*Clinical response*			0.72			
⩾50%	434	78 (74–83)		1		
<50%	309	76 (70–82)		1.1 (0.8–1.5)		

HR=hormonal receptor; LRRFS=locoregional recurrence-free survival

a Log-rank test.

b Likelihood ratio test.

c Factors entered in the multivariate analysis.

**Table 6:** Univariate and multivariate analyses of prognostic factors for locoregional recurrence-free survival for patients treated by breast conservation

			**Univariate analysis**		**Multivariate analysis^a^**	
	***N***	**10-year LRRFS (%)(IC 95%)**	***P* b**	**RR (IC 95%)**	***P* c**	**RR (IC 95%)**
Age (continuous variable)			0.0009	0.96 (0.94–0.98)		
						
*Age (dummy variable)* d			<10^−6^		<10^−5^	
>40	382	77 (72–82)		1		1
⩽40	94	49 (38–63)		2.6 (1.8–3.7)		2.6 (1.7–3.7)
						
*Menopause* d			0.1			
No	353	69 (63–74)		1		
Yes	123	80 (73–88)		0.7 (0.4–1.1)		
						
*Clinical tumour stage* d			0.81			
cT2	391	72 (67–77)		1		
cT3	85	71 (60–83)		1.1 (0.7–1.7)		
						
*Clinical nodal status* d			0.06			
cN0	315	73 (68–79)		1		
cN1	161	68 (60–77)		1.4 (1.0–2.1)		
						
*Histological type* d			0.12			
Ductal	434	72 (68–77)		1		
Lobular	42	62 (47–82)		1.5 (0.9–2.6)		
						
*Hormonal receptors* d			0.54			
HR+	291	72 (66–78)		1		
HR−	114	68 (59–79)		1.1 (0.7–1.7)		
						
*Histological grade*			0.97			
Grade 1	71	73 (63–85)		1		
Grade 2	215	70 (63–77)		1.0 (0.6–1.6)		
Grade 3	156	72 (64–81)		0.9 (0.5–1.6)		
						
*Mitotic index*			0.72			
Grade 1	110	71 (62–83)		1		
Grade 2	125	72 (64–82)		1.1 (0.7–1.8)		
Grade 3	195	70 (63–78)		1.2 (0.8–1.9)		
						
*Clinical response*			0.29			
⩾50%	343	74 (69–79)		1		
<50%	127	67 (58–77)		1.2 (0.8–1.8)		
						
*Locoregional treatment*			<10^−3^			
Postop	308	77 (71–83)				
Preop/excl	168	61 (53–70)		2.0 (1.4–2.8)		

HR=hormonal receptor; LRRFS=locoregional recurrence-free survival

a Multivariate analysis after stratification on local treatment.

b Log-rank test.

c Likelihood ratio test.

d Factors entered in the multivariate analysis.

Preop/excl: locoregional treatment consisted in preoperative or exclusive radiotherapy.

**Table 7:** Univariate and multivariate analyses of prognostic factors for overall survival

			**Univariate analysis**		**Multivariate analysis**	
	***N***	**10-year LRRFS (%)(IC 95%)**	***P***	**RR (IC 95%)**	***P***	**RR (IC 95%)**
Age (continuous variable)			0.7	1.0 (0.99–1.02)		
						
*Age (dummy variable)*			0.11			
>40	597	67 (63–72)		1		
⩽40	153	60 (52–69)		1.3 (0.9–1.7)		
						
*Menopause* a			0.22			
No	563	67 (63–72)		1		
Yes	187	63 (55–71)		1.2 (0.9–1.6)		
						
*Clinical tumour stage* a			0.005			
cT2	556	68 (64–73)		1		
cT3	194	59 (51–67)		1.5 (1.1–1.9)		
						
*Clinical nodal status* a			<10^−7^		<10^−3^	
cN0	465	73 (69–78)		1		1
cN1	285	54 (48–61)		2.0 (1.6–2.6)		2.1 (1.6–2.7)
						
*Histological type*			0.44			
Ductal	672	65 (61–69)		1		
Lobular	78	72 (62–84)		0.8 (0.6–1.3)		
						
*Hormonal receptors (HR)* a			<10^−7^		<10^−3^	
HR+	466	70 (66–75)		1		1
HR−	166	49 (42–58)		2.2 (1.7–2.8)		2.4 (1.8–3.2)
						
*Histological grade* a ^,^ b			0.0004		0.02	
Grade 1	114	78 (69–87)		1		1
Grade 2	362	66 (61–71)		1.8 (1.1–2.8)		1.8 (1.1–2.8)
Grade 3	217	58 (51–66)		2.5 (1.6–3.9)		1.9 (1.2–3.1)
						
*Mitotic index* a			<10^−4^			
Grade 1	182	77 (70–85)		1		
Grade 2	189	67 (60–74)		1.6 (1.1–2.4)		
Grade 3	297	59 (53–65)		2.2 (1.5–3.2)		
						
*Clinical response* a			0.15		0.003	
⩾50%	434	67 (63–73)		1		1
<50%	309	64 (58–70)		1.2 (0.9–1.6)		1.5 (1.2–2.0)

HR=hormonal receptor; LRRFS=locoregional recurrence-free survival

a Factors entered in the multivariate analysis.

b The mitotic index was not entered into the model as it was highly correlated with the histological grade.
